# Microangiopathic Hemolytic Anemia as the Initial Presentation of Metastatic Signet-Ring Cell Carcinoma of the Colon: A Case Report

**DOI:** 10.7759/cureus.76034

**Published:** 2024-12-19

**Authors:** Pradip Chaudhary, Nikky Maharjan, Bhawuk Subedi

**Affiliations:** 1 Internal Medicine, Hurley Medical Center, Flint, USA

**Keywords:** bone metastasis, chemotherapy agents, microangiopathic hemolytic anemia, signet-ring cell carcinoma, thrombotic thrombocytopenic purpura (ttp)-like syndrome

## Abstract

Microangiopathic hemolytic anemia (MAHA) is a condition characterized by intravascular fragmentation of red blood cells, leading to the characteristic finding of schistocytes on a peripheral blood smear. The differential diagnoses of MAHA include thrombotic thrombocytopenic purpura (TTP), hemolytic-uremic syndrome (HUS), disseminated intravascular coagulation (DIC), idiopathic thrombocytopenic purpura (ITP), infections, malignancies, and solid organ transplantation. The commonly associated malignancies with MAHA are gastric, breast, prostate, lung, and lymphoma. Signet-ring cell carcinoma (SRCC) of the colon is a rare form of cancer and is associated with a very poor prognosis. SRCC often presents at an advanced stage, with symptoms such as altered bowel habits, abdominal pain, distension, and potential bowel obstruction or perforation. We report a case of a 51-year-old female who presented with hemolytic anemia, thrombocytopenia, and schistocytes on her peripheral blood smear. In the setting of a high PLASMIC score, she was initially treated for TTP without improvement, warranting further evaluation, and was ultimately diagnosed with SRCC of the colon.

## Introduction

Microangiopathic hemolytic anemia (MAHA) refers to intravascular hemolysis due to narrowed microvasculature (arterioles and capillaries) attributable to various causes. The differential diagnoses of MAHA include thrombotic thrombocytopenic purpura (TTP), hemolytic-uremic syndrome (HUS), disseminated intravascular coagulation (DIC), idiopathic thrombocytopenic purpura (ITP), infection, malignancies, drug-induced (rifampin, chemotherapeutic agents, monoclonal antibodies, opiates), and solid organ transplant [1].

The two most common cancers associated with MAHA are gastric and breast adenocarcinoma, accounting for approximately 26% and 21% of compiled cases, respectively [2]. Cancer-related MAHA (CR-MAHA) is a paraneoplastic syndrome that is characterized by Coombs-negative hemolytic anemia and thrombocytopenia. MAHA is very rare in colon cancer, and only a few cases have been reported, particularly with cancer recurrence [3-5].

Signet-ring cell carcinoma (SRCC) is a histologic variant of adenocarcinoma that accounts for 0.3% to 4.6% of all colorectal cancer cases with poor prognosis. Its presentation varies from symptoms of altered bowel habits to features of bowel obstruction [6]. The studies on treatment and patient outcomes for CR-MAHA are limited; however, some studies have indicated improved outcomes with the treatment of underlying malignancy [7]. Here, we report a case of a 51-year-old woman who presented with MAHA as the presenting feature of metastatic SRCC.

## Case presentation

A 51-year-old woman with a past medical history of type 2 diabetes mellitus, hypertension, hyperlipidemia, and chronic tobacco smoking presented to our hospital for evaluation of altered mental status and abdominal pain for a week. A week prior to admission, the patient was treated with cephalexin for an uncomplicated UTI and a bowel regimen for constipation. Upon presentation, she was awake and alert but confused. The patient also complained of back pain and shortness of breath; however, cardiopulmonary and musculoskeletal examinations were unremarkable without any bony tenderness. In addition, no gross lymphadenopathy was noted.

Her vital signs were within the normal range. Her initial lab workup was notable for anemia (hemoglobin, 9.2 g/dL) and thrombocytopenia. Her renal function was within the normal limit. The inflammatory markers were also elevated: erythrocyte sedimentation rate (ESR) of 42 mm/hour (normal range: 0-30 mm/hour) and C-reactive protein (CRP) of 327.19 mg/L (normal range: 0.0-10 mg/L), which is not typical of MAHA; therefore, further extensive workups were done to evaluate the underlying cause of elevated inflammatory markers.

Further workup showed features suggestive of hemolysis, including an elevated lactate dehydrogenase (LDH) level of 3,078 U/L (normal range: 100-225 U/L), decreased haptoglobin level of 125 mg/dL (normal range: 16-200 mg/dL), elevated total bilirubin level of 1.8 mg/dL (normal range: 0.3-1.2 mg/dL), normal indirect bilirubin level of 0.7 mg/dL, and reticulocyte percentage of 2.5%.

Subsequently, during the course, platelet count steadily declined from 145 k/UL to 115 k/UL, then to 87 k/UL, followed by 33, 22, and eventually <5 k/UL, without any overt signs of bleeding. The hemoglobin level also decreased from 12.1 to 9.2 g/dL and then to 6.8 g/dL. A peripheral blood smear revealed schistocytes and atypical lymphocytes. Hepatitis panel, HIV serology, serotonin release assay, heparin-induced platelet antibody, folic acid, vitamin B12, and ANA/anti-dsDNA were all unremarkable. She was treated with plasmapheresis daily for a week, methylprednisolone 1 g daily for three days, followed by 500 mg for four days, rituximab, and caplacizumab daily for a week due to her high risk for TTP in the setting of a high PLASMIC score of 6.

**Figure 1:**
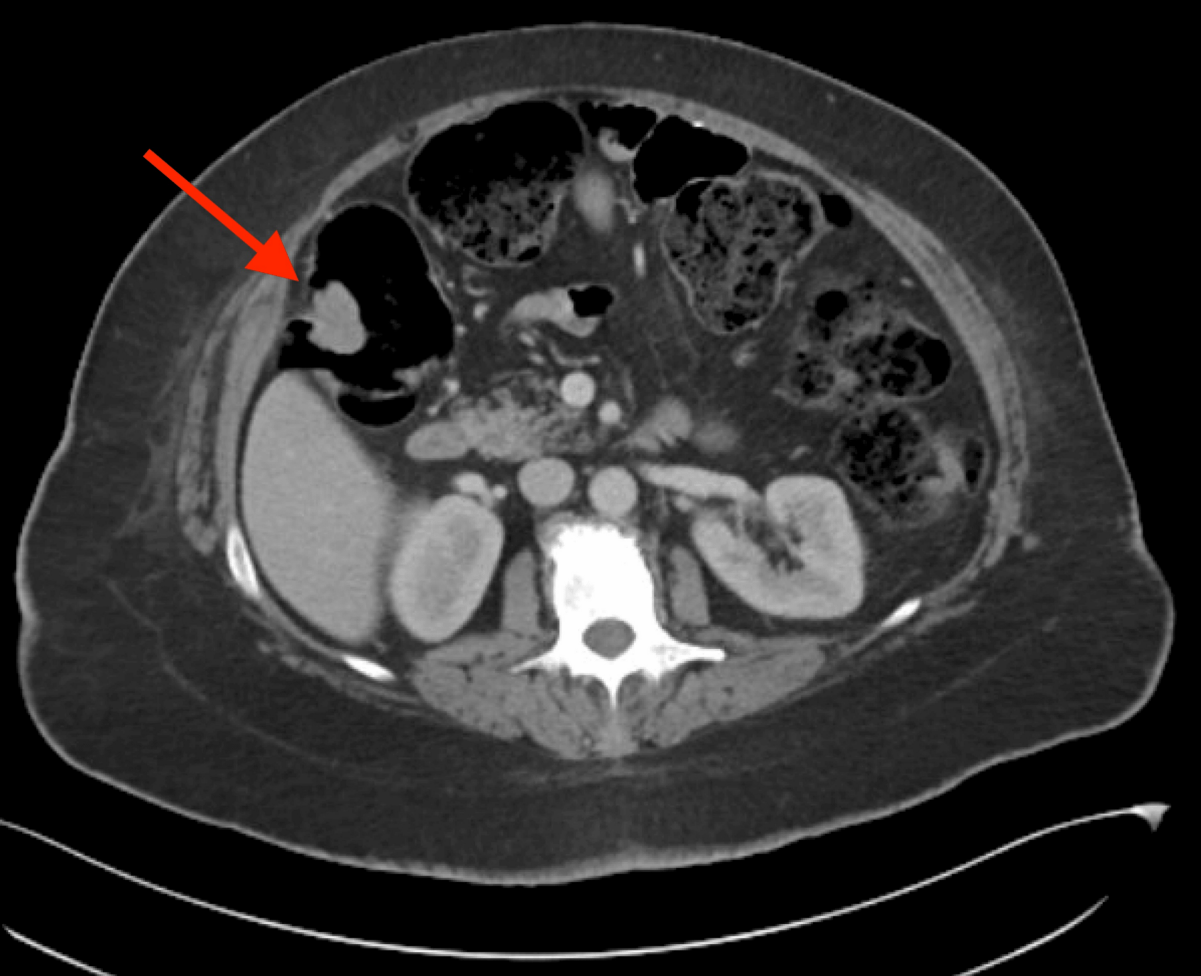
The CT scan of the abdomen and pelvis revealed a lobulated polypoid lesion in the hepatic flexure, measuring 2.9 x 1.7 x 2.8 cm (red arrow).

However, she remained refractory to treatment with worsening thrombocytopenia (platelets <5,000) and anemia (hemoglobin 6.1 g/dL), requiring multiple units of packed RBC and platelet transfusions. The coagulation profile revealed PT/INR (prothrombin time/international normalized ratio) of 14.5 seconds/1.47, APTT (activated partial thromboplastin time) of 25.8 seconds, fibrinogen of 454 mg/dL, and D-dimer >10 mg/L. The ADAMTS13 level was later found to be greater than 30%, making a diagnosis of TTP unlikely (Table 1). On day two, a CT scan of the abdomen and pelvis was performed due to persistent abdominal pain, which revealed a lobulated polypoid lesion in the hepatic flexure measuring 2.9 x 1.7 x 2.8 cm (Figure 1).

**Table 1:** Lab values with reference range on initial presentation. WBC: white blood cells; RBC: red blood cells; LDH: lactate dehydrogenase; ESR: erythrocyte sedimentation rate; CRP: C-reactive protein; INR: international normalized ratio; APTT: activated partial thromboplastin time; CEA: carcinoembryonic antigen; CA: carbohydrate antigen

Parameters	Results (On Presentation)	Reference Range
WBC	6	4.0–11 k/µL
RBC	3.10	4.0–5.2 m/µL
Hemoglobin	9.2	13.5–17.5 g/dL
Platelets	115	130–430 k/µL
Total Bilirubin	1.8	0.3–1.2 mg/dL
Indirect Bilirubin	0.7	0.2–0.8 mg/dL
LDH	3078	100–225 µ/L
Haptoglobin	125	16–200 mg/dL
Reticulocytes %	2.5	0.5–2.0%
ESR	42	0–30 mm/hour
CRP	327.19	0–10 mg/L
PT	14.5	9.2–11 seconds
INR	1.47	0.8–1.1
APTT	25.8	22–32 seconds
Fibrinogen	454	193–473 mg/dL
D-Dimer	>10	0–0.49 mg/L
CEA	76.4	0–5 ng/mL
CA 19-9	42.5	≤37 µ/mL
CA 125	41.9	0–35 µ/mL
ADAMTS13 Activity	>30	>30%

**Figure 2:**
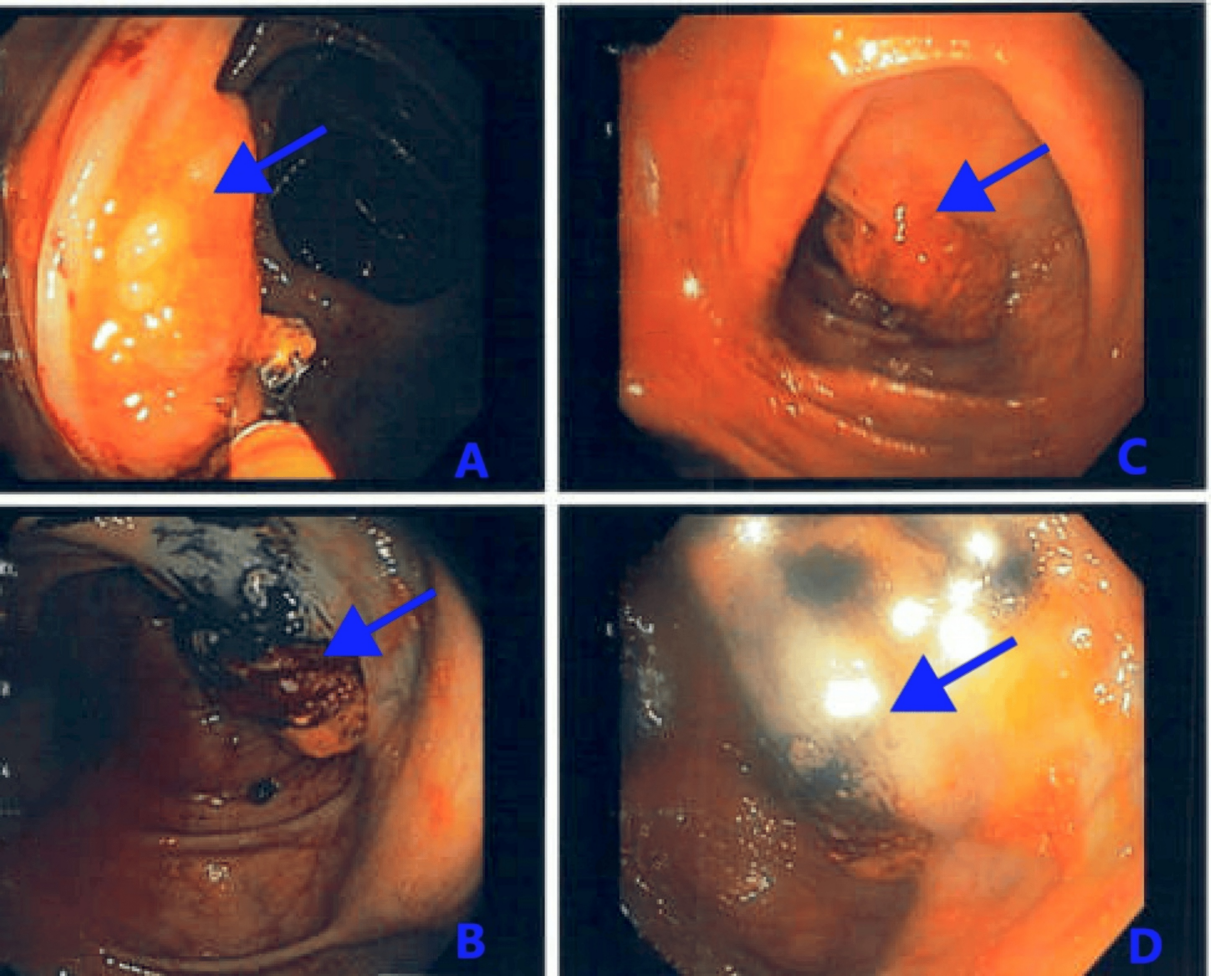
A colonoscopy demonstrating an ulcerative mass (blue arrows) along the transverse colon in different views (A–D).

Tumor markers were significantly elevated, with a carcinoembryonic antigen (CEA) level of 76.4 ng/mL (normal range: 0.0-5 ng/mL), carbohydrate antigen-125 (CA-125) of 41.9 U/mL (normal range: 0.0-30.2 U/mL), and carbohydrate antigen-19-9 (CA-19-9) of 42.5 U/mL (normal range: ≤35 U/mL). A colonoscopy demonstrated an ulcerative mass along the transverse colon (Figure 2), and a biopsy was taken, which revealed SRCC of the colon.

A bone marrow biopsy was also performed, demonstrating a micro-cluster of signet-ring cells in the background of an extensively necrotic, cytokeratin-positive tumor, consistent with bone marrow involvement by SRCC of the colon. An exploratory laparotomy with a right colectomy was deferred due to poor surgical candidacy. She was subsequently started on chemotherapy with the FOLFOX regimen (leucovorin, fluorouracil, and oxaliplatin) for two days, followed by two cycles. The cell counts decreased on the initiation of chemotherapy: WBC 1.1 k/µL, RBC 2.27 m/µL, and platelet count 10 k/µL, which significantly improved after the therapy. The cell counts post-therapy were as follows: WBC 9.3 k/µL, RBC 3.5 m/µL, and platelet count 28 k/µL.

## Discussion

TTP, Shiga toxin-mediated HUS (ST-HUS), drug-induced thrombotic microangiopathy (DITMA) syndromes, and complement-mediated TMA (CM-TMA) are common primary thrombotic microangiopathy (TMA) syndromes that present with MAHA and thrombocytopenia [8]. These syndromes are treated based on the pathophysiology of TMA. Systemic diseases such as systemic infection, severe hypertension, severe preeclampsia, systemic lupus erythematosus, and malignancies are common secondary causes of MAHA [8]. Gastric cancer is the most common cancer causing MAHA, followed by breast, prostate, and lung cancers [9].

CR-MAHA is a rare paraneoplastic syndrome, particularly seen in patients with solid tumors [10]. However, MAHA could be the only presenting symptom of malignant cancers. In our case, MAHA was the initial finding, which later led to the diagnosis of metastatic SRCC of the colon. CR-MAHA usually indicates the late stage of cancer with a poor prognosis. In one case series of 168 patients with CR-MAHA, 46.5% mortality was noted within a month with or without treatment. However, rapid and near-complete remission of the hematological parameter MAHA was noted after a single cycle of chemotherapy in our patient, which was also seen in a few cases reported in the literature [10].

The pathophysiology behind CR-MAHA is still unknown; however, three mechanisms have been proposed in the literature: (a) embolization of tumors in the microvasculature, such as arterioles and capillaries, leading to red blood cell lysis; (b) endothelial cell activation due to circulating cancer cells, leading to an inflammatory cascade; and (c) secretion of mucin by tumor cells, von Willebrand factor (vWF) by bone marrow metastasis, and tissue factor expression by tumor and endothelial cells, which collectively lead to activation of the coagulation pathway [11]. Most of the cases of MAHA due to cancer have normal or mildly reduced ADAMTS13 levels compared to primary TTP, in which ADAMTS13 levels are markedly reduced. Even though the PLASMIC score was high in our case, the ADAMTS13 level was over 20%, which led us to consider other causes of MAHA besides TTP.

SRCC is a rare histological variant of adenocarcinoma, with the most common sites being the stomach, colon, esophagus, rectum, and lungs in descending order [12]. Only 1% of colon cancer cases comprise SRCC [13]. SRCC of the colon tends to present at an early age, with a mean age of onset 3.5 years earlier compared to nonvariant adenocarcinoma of the colon. SRCC of the colon is usually diagnosed at the metastatic stage with poor tumor grade and has a poor prognosis with a median survival of only 21.6 months [12]. During the presentation, metastases to the local lymph nodes, peritoneal surfaces, ovaries, lungs, and bone marrow frequently make SRCC more difficult to treat. In our patient, extensive necrosis of myeloid tissue and medullary stroma, along with clusters of signet-ring cells, was noted in the bone marrow biopsy, indicating bone marrow metastasis of SRCC of the colon. There are multiple reported cases of MAHA in patients with SRCC of the stomach and breast, as well as cases of unknown origin. However, to the authors' knowledge, no cases of MAHA due to SRCC of the colon have been reported in the literature to date.

Although patients with primary TMA and MAHA have comparable clinical signs and laboratory results owing to other conditions, patients with CR-MAHA are more likely to experience bone pain and dyspnea, which was also observed in our case [3]. It is important to rule out the underlying cause of MAHA, as this helps tailor the treatment approach in a timely manner and avoid unnecessary treatment.

In an acute clinical setting with a high PLASMIC score, it is difficult to differentiate TTP from CR-MAHA, so patients are often started on plasma exchange (PE) therapy [14]. Normal or near-normal ADAMTS13 activity, inadequate response to PE therapy, bone pain, and dyspnea in a patient with TTP features should be used as clues for the diagnosis of CR-MAHA. Hence, if these features are present, the patient should also be evaluated for underlying malignancy.

A bone marrow biopsy could be an effective diagnostic tool to find out the underlying cause of suspected CR-MAHA patients without a known malignancy, as most CR-MAHA cases have bone marrow metastasis [5]. Once the diagnosis of CR-MAHA is made, appropriate chemotherapy should be started without delay, along with symptomatic management with RBC and platelet transfusion, as it is the most reliable treatment option.

## Conclusions

Microangiopathic hemolytic anemia could be the presenting feature of an underlying malignancy and can be misdiagnosed as TTP. A normal ADAMTS13 level, a poor response to PE therapy, and pulmonary and bone symptoms should prompt the evaluation for CR-MAHA. Early detection and efficient chemotherapy can cause hematologic remission and avoid PE therapy.
